# Simplified ICP OES-Based Method for Determination of 12 Elements in Commercial Bottled Birch Saps: Validation and Bioaccessibility Study

**DOI:** 10.3390/molecules25051256

**Published:** 2020-03-10

**Authors:** Maja Welna, Anna Szymczycha-Madeja, Pawel Pohl

**Affiliations:** Division of Analytical Chemistry and Chemical Metallurgy, Faculty of Chemistry, Wroclaw University of Science and Technology, Wybrzeze Wyspianskiego 27, 50-370 Wroclaw, Poland; maja.welna@pwr.edu.pl (M.W.); pawel.pohl@pwr.edu.pl (P.P.)

**Keywords:** birch sap, alternative sample preparation procedures, straightforward multi-element analysis, inductively coupled plasma optical emission spectrometry, validation, bioaccessibility study, Recommended Daily Intakes

## Abstract

Commercially bottled birch saps (BSs) were analyzed for several nutrient (Ca, Cu, Fe, Mg, and Zn) and toxic (As, Cd, Ni, and Pb) elements using inductively coupled plasma optical emission spectrometry (ICP OES). The method was validated under the conditions of several sample preparation procedures, including a traditional digestion as well as alternative non-digestion schemes. It was found that the direct analysis of untreated BSs gives the best results, i.e., limits of detection at 0.02–5.8 ng mL^−1^, precision better than 5%, accuracy from 98.0% to 104.5% and determination of 12 elements in a short time (~1 min per sample). The multi-element analysis of nine commercially available bottled BSs showed that they contained mainly Mg and Ca, small quantities of Mn, Zn, Cu, and Fe, but are free from toxic elements such as As, Cd, Ni, and Pb. Additionally, the nutritional value of BSs was examined using in vitro gastro-intestinal digestion (GID) to determine the bioaccessible fraction of elements. Accordingly, bioaccessibility of nutritious ones (Ca, Cu, Fe, Mg, Zn) was <40%. Drinking daily 1 L of BSs covered <2.5% of recommended dietary intakes (RDIs) of the aforementioned elements. Only the bioaccessibility of Mn highly contributes to its RDI.

## 1. Introduction

The birch sap (BS) collected from *Betula pendula* species, is widely acclaimed for its nutritional, antioxidative and curative properties [1,2,3,4,5,6,7]. It can be consumed for the whole year fresh or as a naturally fermented, refreshing and cooling, low-alcohol drink [7,8,9,10,11]. It can also be used as a raw material for the production of commercially available beverages, like bottled birch saps [7,8,9,10,11]. Apart from pleasant sweetish taste, BS is considered to be today’s “health elixir”, i.e., a rich source of carbohydrates, proteins, organic and amino acids, vitamins (C, group B), antioxidants (flavonoids), phenolic compounds, tannins, as well as important nutrients, namely Ca, Na, Mg, K, Fe, Mn, Zn, and Cu [5,6,8,11,12,13]. All these constituents positively influence the human organism, and hence, the incorporation of BSs into a daily diet instead of synthetic and artificial food ingredients is highly valuable [5,6,8,11,12,13].

Among all substances contained in the BS, its elemental composition is treated as a key parameter promoting beneficial effects on human health [4,14]. In this context, total concentrations of elements are commonly compared with their nutritional standards, including Recommended Daily Intakes (RDIs) for adults, and the dietary benefits of drinking 1 L of the BS per day are evaluated [4,11,14]. It is frequently assumed that elements are 100% available and absorbed by the body during intraoral digestion. In fact, actual nutritional benefits to human health associated with drinking of the BS can be assessed only after an estimation of the bioaccessibility of elements. In this case, an in vitro digestibility model with artificial enzymes can be used to mimic physicochemical and enzymatic processes in the human gastro-intestinal tract [15,16]. Consequently, the determination of total element contents may lead to overestimation of the coverage of their RDIs. Unfortunately, no papers have been devoted to the bioaccessibility study of elements in BSs. For that reason, a reliable judgment of the potential harm to human health associated with the intake of BSs and the elements included in them deserves attention.

The health-promoting potential of the BS can be problematic because its elemental content is highly variable and strongly depends on environmental and anthropogenic factors as well as individual physicochemical properties of particular trees and their geographical locations [4,6,8,12,14]. Therefore, exact knowledge about the elemental content of consumed BSs, in reference to both nutritionally essential and toxic elements, is needed to assess their quality and safety, but should be conducted using properly validated spectrometric methods.

Surprisingly, information about the elemental composition of BS is limited to the raw material, i.e., freshly collected samples. Such information is obtained by applying flame or graphic furnace atomic absorption spectrometry (F-AAS or GF-AAS) or inductively coupled plasma optical emission spectrometry (ICP OES), which are normally combined with previous sample treatment based on wet digestion in concentrated acids [4,5,6,8,10,11,12,14]. A little effort is taken to develop simple and fast methods, i.e., with no or only with minimum sample preparation prior to spectrometric measurements. Such methods could be adequate for the reliable determination of various elements in bottled BSs, bringing a quick answer about quality and safety of consumed birch beverages. Only few studies have been carried out so far to analyze the fresh BS based on the content of selected elements where simplified sample preparation was used [5,6,14]. Unfortunately, reported methods [5,6,14] were not validated, hence their metrological quality can be questioned.

For this reason, the main objective of this work was to develop and validate a simple and fast ICP OES method for determination of total concentrations of 12 elements (As, Ca, Cd, Cr, Cu, Fe, Mg, Mn, Ni, Pb, Sr, and Zn) in bottled BSs. In reference to this, six different sample treatments (P1–P6), including a traditional wet digestion in concentrated HNO_3_ (P1), and several alternative procedures, i.e., acidification with concentrated HNO_3_ to 1% (*v*/*v*) (P2) and 2% (*v*/*v*) (P3), two-fold dilution with 2% (*v*/*v*) (P4) and 10% (*v*/*v*) HNO_3_ (P5), and no treatment direct analysis (P6), were tested. Procedures were compared in terms of trueness and precision of results, as well as limits of detection (LODs) of elements achievable with ICP OES. Finally, the optimal alternative procedure was applied for rapid multi-element analysis of nine bottled BSs marketed in Poland to evaluate their quality and safety. To check nutritional values of these products, contributions to RDIs for particular elements, related to the intake of one liter of BS, were calculated based of their bioaccessibility. Hence, the second objective of this work was to evaluate the bioaccessibility of studied elements from analyzed BSs after the application of gastrointestinal digestion (GID).

## 2. Results and Discussion

### 2.1. Strategy to Evaluation of Simplified Method of Analysis

As the main goal of this work was to develop and validate the method for multi-element analysis of BSs by ICP OES with no elaborate previous sample treatment, special attention was paid to the sample preparation step prior to spectrometric measurements. Our intention was to find such an alternative procedure that eliminates usual acid-based wet digestion with concentrated oxidative reagents at elevated temperatures on the one side, and guarantees appropriate trueness, precision, and sensitivity for the dependable ICP OES determination of total concentrations of 12 elements in BSs on the other side. At first, the performance of ICP OES combined with tested procedures (P1–P6) was evaluated by examining important validation parameters, i.e., slopes (a), determination coefficients (R^2^) and linearity ranges of calibration curves, LODs of elements, and precision (expressed as relative standard deviation, %RSD) of repeated measurements of analytes responses. Next, the suitability of alternative sample preparation procedures (P2–P6) of BSs prior to their multi-element analysis by ICP OES was verified by analyzing the selected BS, i.e., BS9.

For this purpose: (1) standard solutions were prepared using simple aqueous (P6) and matrix-matched standard solutions (P1–P5) of analytes within the concentration range of 0.01–5.0 µg mL^−1^; (2) then, six external calibration curves, each containing 9 points, were plotted; (3) after that, BS9 was treated using P1–P6 and analyzed using ICP OES, and concentrations were calculated using previously obtained external calibration curves; (4) finally, the obtained concentrations from P2–P6 were compared with those from P1 (the reference method); (5) validity of the obtained results from P1 itself was verified in two ways. The first one based on analysis of two Certified Reference Materials (CRMs) and the second one on spike-and-recovery experiments carried out for the BS9. The significance of differences between results achieved with reference (P1) and alternative (P2–P6) sample preparation procedures was established using appropriate statistical tests, namely the one-tailed Snedecor–Fisher *F*- and the two-sample Student *t*-tests [17]. Additionally, analogously as for the procedure P1, trueness of results obtained with all alternative procedures (P2–P6) was checked by carrying out spike-and-recovery experiments for three different concentrations of elements added to BS9 samples (depending on their concentrations determined in the BS9).

### 2.2. Method Development

#### 2.2.1. Effect of the Sample Preparation Procedure on Analytical Characteristic of ICP OES

Using matrix-matched standards, the effect of the HNO_3_ concentration, remained in final solutions from P1–P5 procedures, was examined on determination of 12 elements by ICP OES. In case of the procedure P6, simple aqueous standards were considered. It was established that independently of the sample procedure used (P1–P6), calibration curves for all studied elements were linear within their concentration ranges. Superior determination coefficients were also established (R^2^ ≥ 0.999). Precision for replicated (*n* = 3) measurements of analytes signals was good and did not exceeded 3% (RSDs between 0.1–2.8%). Similarly, comparing slopes of calibration curves it was found that differences between their values were minor (1–5%). Measuring respective blanks, LODs of elements were evaluated. LODs were calculated as concentrations (in ng mL^−1^) corresponding to three standard deviations (3 × SD) of 10 measurements of blank solutions (3 σ criterion). Additionally, taking into account sample portions and final dilution factors employed in compared sample preparation procedure, these LODs (abbreviated as method LODs) were referenced to analytes concentrations (in ng mL^−1^) in original samples. LOD values for procedures P1–P6 are listed in Table 1.

As can be seen from Table 1, LODs of elements for procedures P1–P5 were at the same level (0.050–6.5 ng mL^−1^), or a little bit higher than those assessed using the procedure P6 (0.020–5.8 ng mL^−1^). Exceptions were Cr, Fe, Mg, Sr, and Zn. For these elements, the highest LODs were obtained using procedure P1 (1.6–25 ng mL^−1^). This was mostly due to higher values of the procedural blank attained for P1 as compared to those determined in remaining procedural blanks (P2–P6). Among all sample treatments, detectability of direct analysis (P6) was the lowest. This was applicable to all 12 elements determined by ICP OES. In contrast, differences in LODs between procedures were evident for the case when these values were established for the original sample (method LODs). In general, detectability of elements decreased in the following order: P6 > P2~P3 > P4~P5 > P1. It was mainly caused by dilution of original sample used in particular sample preparation procedures, i.e., four-fold (P1), ~2% (P2), ~11% (P3), 2-fold (P4, P5) and 0 (P6). Undoubtedly, the method of measuring LODs of elements should be taken into account as a large dilution of the original BS samples may lead to non-detection of low concentrated analytes in prepared sample solutions. In view of this, the procedure P6 (direct analysis) was shown to be favorable treatment, particularly for (ultra)trace analysis.

#### 2.2.2. Effect of the Preparation Procedure on Reliability of Analytical Results

Subsequently, the suitability of non-digestive preparation procedures (P2–P6) of BSs prior to their multi-element analysis by ICP OES was verified. The effect of tested alternative sample preparation procedures on trueness of results of ICP OES analysis was examined in relation to their closeness to reference values, achieved using the procedure P1. Total concentrations of 12 elements (mean values along with standard deviations (SDs, *n* = 3)) determined by ICP OES in differently prepared sample solutions of the BS9 were used. Initially, reliability of the reference procedure (P1) was verified by analyzing two CRMs, i.e., INCT-TL-1 (black tea leaves) and NCS-ZC73036 (green tea). It was established that determined concentrations of all elements well corresponded to certified values at the 95% confidence level (*p* = 0.05), as shown using the Student *t*-test [17]. Accordingly, calculated values of this test (*t*_calculated_) for all elements were lower than the critical value (*t*_critical_) equal to 4.303. It confirmed that the chosen sample preparation procedure (P1) guarantied reliable results of multi-element ICP OES analysis and could be treated as the reference sample treatment.

Before comparing the mean concentrations of studied elements obtained with P2–P6 procedures with those obtained using the reference procedure (P1), the one-tailed Snedecor–Fisher *F*-test with a critical value (*F*_critical_) of 19.00 (*p* = 0.05) was used to examine significant differences between SDs of these means [17]. When calculated values of the *F*-test (*F*_calculated_) were lower than the *F*_critical_ value (*F*_calculated_ < *F*_critical_), what indicated that precision of compared results was at the same level, the two-sample Student *t*-test with a critical value (*t*_critical_) of 2.776 (*p* = 0.05) was used [17]. Otherwise, i.e., in case when *F*_calculated_ > *F*_critical_, the Cochran–Cox *C*-test was used with a critical value (*C*_critical_) of 4.303 (*p* = 0.05) [17]. Results of multi-element analysis of the BS9 by ICP OES (total concentrations of 11 elements given as mean values) combined with different sample preparation procedures (P1–P6) are presented in Table 2. Table 3 presents *F*_calculated_ and |*t*_calculated_| values. Additionally, the precision of results (as %RSD) was calculated.

As can be seen from Table 2, concentrations of As, Cd, Ni and Pb were below their respective LODs. Similarly, the reference procedure (P1) failed regarding Cr. It could be determined, however, only by using alternative sample preparation procedures (P2–P6). Such behavior was suspected, taking into account the (ultra)trace amount of Cr in the BS9 (~5 ng mL^−1^) and the dilution factor (4-fold) applied in the procedure P1. This clearly pointed out the necessity for the evaluation of novel sample treatments, particularly for the determination of trace amounts of elements by spectrometric methods. Therefore, the verification of validity of results of element analysis of the BS9 by ICP OES combined with alternative sample preparation procedures (P2–P6) was limited to seven out 12 elements, i.e., Ca, Cu, Fe, Mg, Mn, Sr, and Zn (see Table 3).

It was established that calculated values of the *F*-test were lower than its critical value (*F*_calculated_ < *F*_critical_), indicating that differences between SDs of results obtained using the reference procedure (P1) and alternative sample preparation procedures P2–P6 were insignificant. In these cases, the *t*-test was used to test the significance of differences between mean concentrations of elements. Few exceptions were found, i.e., *F*_calculated_ > *F*_critical_, and included Ca, Mn and Zn (P3) and Mn (P2). In these cases, the *C*-test was used, and calculated values of this test are listed in Table 3 as well.

Considering RSDs (Table 2), precision using the reference sample preparation procedure (P1) was within 0.68–4.2% with average of 3.2%. For other non-digestive procedures (P2–P6), RSDs (along with their average values in brackets) were as follows: P2: 0.64–3.6% (1.6%), P3: 0.28–11% (2.4%), P4: 1.5–16% (4.5%), P5: 0.78–14% (3.2%) and P6: 0.68–4.1% (1.9%). Comparing these results, it was stated that no treatment (direct analysis, P6) and acidification to 1% (*v*/*v*) with concentrated HNO_3_ (P2) provided the best precision of results obtained for the studied elements.

Considering the trueness of results, it appeared that only direct analysis of the untreated BS9 samples (P6) gave mean concentrations of studied elements consisted with those obtained using the reference procedure (P1). Interestingly, in case of P2–P5 procedures, it was observed that mean concentrations of studied elements were lower than these determined with the procedure P1 and decreased with an increase in the concentration of HNO_3_ in prepared sample solutions. The following descending order of mean concentrations could be arranged: P6 > P4 > P2 > P5 > P3. Adequateness of direct analysis (P6) was proved by using *F*- and *t*-tests. According to these significance tests, direct analysis (P6) of BSs provided as precise and true results for all 7 elements as those obtained with the reference procedure (P1). Dilution of the BS9 with 2% (*v*/*v*) HNO_3_ (P4) resulted in trueness of a lower number of elements. Statistically insignificant differences between mean concentrations were established only for three (Mn, Sr and Zn) out of seven elements. Unfortunately, dilution of the BS9 with 10% (*v*/*v*) HNO_3_ (P5), as well as its acidification to 1% (*v*/*v*) (P2) and 5% (*v*/*v*) (P3) with concentrated HNO_3,_ were found to be useless and could be responsible for biased results.

Additionally, the spike-and-recovery experiment with three different concentration levels was made for the BS9. In these experiments, both detectable elements (Ca, Cr, Cu, Fe, Mg, Mn, Sr, and Zn) and those with concentrations established previously below their LODs (namely As, Cd, Ni, and Pb) were considered. The level of additions depended on mean concentrations of elements determined in the BS9 (see Table 2), and hence ranged from 0.10 to 0.50 µg mL^−1^ (in final sample solution) for Ca, Fe, Mg, Mn, Sr, and Zn, and from 0.010 to 0.050 µg mL^−1^ (in final sample solution) for As, Cd, Cr, Cu, Ni, and Pb. In the case of Ca and Mg, additions were made to appropriately diluted BS9 samples. Recoveries of added elements were calculated by analyzing unspiked and spiked BS9 samples. Results are given in Table 4.

Obtained recovery values corresponded well with outcomes of the statistical analysis, i.e., *t*- and *C*-tests. Accordingly, results for the reference procedure (P1) and direct analysis of BS9 (P6) were reliable, i.e., quantitative recoveries of all elements were achieved, independently of the spike level. Recoveries were as follows: 96.9–109% (P1) and 98.0–104% (P6). Importantly, in case of direct analysis (P6), these results were the evidence of absence of any interfering effects coming from undecomposed sample matrix constituents of the BS9. This pointed out that direct analysis (P6) gave dependable results for all studied elements and could be alternatively used instead of the microwave-assisted wet digestion procedure (P1) before multi-element analysis of BSs by ICP OES. For other sample preparation procedures (P2–P5) recoveries of added elements were poorer, i.e., 92.0–129% (P2), 87.5–121% (P3), 82.9–124% (P4) and 76.6–116% (P5).

To sum up, considering the important validation parameters investigated here, particularly the trueness of mean concentrations of elements and their LODs, the direct analysis of bottled BSs (P6) was found to be the most advantageous prior to their multi-element analysis by ICP OES. Consequently, it was used for further studies related to the demonstration of the analytical application of this procedure.

### 2.3. Analytical Application

The proposed no treatment sample preparation procedure (P6) for multi-element analysis by ICP OES was applied to determination of As, Ca, Cd, Cr, Cu, Fe, Mg, Mn, Ni, Pb, Sr, and Zn in 9 bottled BSs. Results of total concentrations of 12 elements (in µg mL^−1^), means (*n* = 3) along with %RSDs in the brackets are presented in Table 5. Additionally, average concentrations of elements within group of BSs along with coefficients of variance (%CVs) are included in this table.

It was observed that all analyzed BSs did not contain As, Cd, Ni and Pb. Concentrations of these elements were below their respective LODs, i.e., 5.8, 0.31, 1.5, and 3.1 ng mL^−1^. Concentration of Cr was either lower than its LOD (0.74 ng mL^−1^) in three BSs (BS2, BS5, BS6). Considering remaining elements, Ca and Mg were minor elements, while Cr, Cu, Fe, Mn, Sr and Zn-trace elements of BSs. Mean concentrations of both groups of elements changed as follows: Ca > Mg > Mn > Fe > Zn > Sr > Cu > Cr. Mean concentrations of Ca and Mg were the highest (12–50 µg mL^−1^), however the level of Ca was four times higher than that of Mg. Both elements were also characterized by the lowest discrepancy of results within the group of BSs as values of CVs were from 13% (Ca) to 23% (Mg). Except for Mn, mean concentrations of other trace elements, i.e., Cr, Cu, Fe, Mn, Sr, and Zn, were low (<1 µg mL^−1^, Fe, Sr, Zn) or very low (<0.01 µg mL^−1^, Cr and Cu). Manganese was present in higher concentration (~3 µg mL^−1^ on average). Importantly, the determined content of this metal in the BS8 (6.28 µg mL^−1^) well corresponded to the value declared by the producer, i.e., 6.30 µg mL^−1^. Oppositely to Ca and Mg, mean concentrations of practically all trace elements varied significantly among all analyzed BSs. Established CVs were high and changed from 65% (Fe) to 103% (Cu). Much lower variation of results was noticed only for Sr (the CV of 18%).

Regarding the literature available, element analysis of BSs concerned only freshly collected BSs [4,5,6,8,10,11,12,14] and focused mainly on determination of concentrations of selected nutrients and toxic elements, i.e., Ca, Mg, K, Mn, Na, Cu, Fe, Zn, Ni, P and Cd. This present work reports for the first time multi-element (12) analysis of commercially available bottled BSs. Nevertheless, considering the mean concentrations of elements determined in BS1–BS9, they matched very well with the concentration ranges reported by other authors for fresh uncommercial BSs, i.e., Ca (5.5–212 µg mL^−1^) [4,5,6,8,11,12,14], Cd (n.d.—7.8 µg L^−1^) [10], Mg (2.9–25 µg mL^−1^) [4,5,6,8,11,14], Mn (0.37–11.3 µg mL^−1^) [5,6,8,12], Cu (n.d.—390 µg L^−1^) [4,5,6,11,12,14], Zn (0.014–4.5 µg mL^−1^) [4,5,6,8,11,14] and Ni (n.d.—0.030 µg mL^−1^) [12]. The only exception was for Fe, whereby the mean concentration of this element established in the present work was nearly four times higher that reported by others (n.d.—0.25 µg mL^−1^) [5,6,8,12,14].

### 2.4. Bioaccessibility Studies

Despite the determination of total concentrations of elements in BSs, in this work, for the first time, their speciation in terms of bioaccessibility was also considered to achieve a better understanding of the nutritional benefits of BSs. The bioaccessibility of elements present in BSs (Ca, Cu, Cr, Fe, Mg, Mn, Sr, and Zn) was evaluated using the optimized and fully validated methodology based on the in vitro gastrointestinal digestion (GID) procedure with artificial fluids proposed in our recent work for infusions of ground and instant coffees [15]. Knowing the total concentrations of studied elements in BSs, determined using the simplified method of multi-element analysis of BSs by ICP OES developed herein, percentage contributions of the bioaccessible fraction of elements were also calculated. The following formula was used: $\frac{A}{C_{t}}\times 100$, where *A* is the concentration of an element determined in the dialyzable fraction of BSs and *C_t_* is its total concentration determined in these BSs. Results of this analysis are given in details in Table 6.

To confirm the trueness of results obtained by the in vitro GID procedure, a mass balance study was carried out. For each element, the sum of its concentrations in dialyzable and non-dialyzable fractions distinguished in analyzed BSs was compared with its total concentration determined in these BSs, and expressed as recovery (in %) (Table 7).

As shown in Table 7, with exception of Cr, quantitative recoveries for remaining elements were achieved, i.e., 101.4–103.9% for Ca, 96.7–108.3% for Cu, 98.4–103.7% for Fe, 100.4–104.2% for Mg, 97.7–102.6% for Mn, 99.5–103.7% for Sr and 100.0–104.1% for Zn. Moreover, the precision of measurements for these elements was also good (RSDs < 5%), changing in the following ranges: 1.3–3.6% (Ca), 2.6–4.9% (Fe), 1.4–3.5% (Mg), 1.1–3.1% (Mn), 1.5–3.5% (Sr) and 0.8–4.3% (Zn). Only for Cu was precision slightly worse, i.e., 2.3–7.1%. In the case of Cr, its concentrations in both fractions (dialyzable and non-dialyzable) were found to be below their respective LODs, i.e., 1.5 and 3.3 ng mL^−1^.

Mean values of contributions of the bioaccessible fraction of studied elements from BSs were within 15.7–37.5% (Table 6). It was found that Ca and Sr were the most bioaccessible elements; contributions of their bioaccessible fraction ranged from 33.9% to 44.9% (with mean of 37.5% and CV of 8.3%) and 28.4–42.9% (with mean of 36.9% and CV of 12%), respectively. Magnesium and Mn had slightly lower bioaccessibility; contributions of their bioaccessible fraction were 30.2–42.0 (with mean of 35.1% and CV of 13%) and 23.0–40.3 (with mean of 31.4% and CV of 17%), respectively. In case of Zn and Cu, their bioaccessibility did not exceed 35% and varied between 24.0–33.7% (with mean of 28.2% and CV of 13%) and 17.6–28.1 (with mean of 22.4% and CV of 15%), respectively. Finally, in the group of all studied elements, the lowest contribution of the bioaccessible fraction was established for Fe, i.e., 13.3–19.7% (with mean of 15.7% and CV of 15%). However, this was justified, because Fe in plant-based products (like the birch tree sap) exists in the non-heme form, which is characterized by quite low bioaccessibility. In addition, other minerals, such as Ca and Zn, may also inhibit bioaccessibility of this metal from the BS. Interestingly, oppositely to total concentrations of studied elements, that, with exception of Ca and Mg, varied significantly within the studied group of BSs (CVs within 65–103%), variation of contributions of the bioaccessible fraction of all elements was low. Established CVs were below 20%, i.e., 8.3%(Ca)—17% (Mn), which indicated that the bioaccessibility of elements from BSs is quite comparable.

Finally, taking into account mean concentrations of essential elements (Ca, Cu, Fe, Mg, Mn, Zn) in the bioaccessible fraction of BSs, the nutritional context of BSs for adults (male and female) was examined in reference to dietary benefits of the intake of 1 L of examined beverages per day. Accordingly, concentrations of these elements were compared with their nutritional standards, including RDIs, i.e., recommended dietary allowances and adequate intakes for male and female in the 31–50-year life stage group, defined by the National Research Council [18]. Respective RDIs (in mg day^−1^) were as follows: 1000 (Ca), 0.9 (Cu), 8–18 (Fe), 420–320 (Mg), 2.3–1.8 (Mn) and 11–8 (Zn).

**Figure 1:**
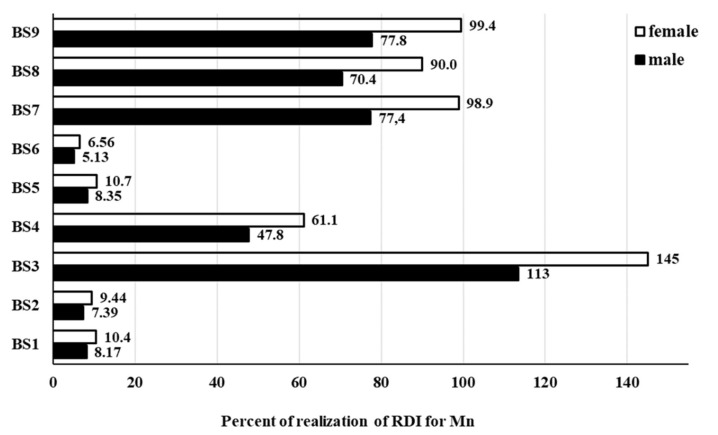
Contribution of birch saps to the Recommended Daily Intakes (RDI) of Mn for male and female related to daily consumption of 1 L of all analyzed birch saps (BS1–BS9).

One L of BSs was established to slightly cover (<2.5%) daily Ca, Cu, Fe, Mg and Zn requirements. RDI standards were covered in 1.9% for Ca, 2.4% for Cu, 1.8% and 0.78% for Fe (for male and female, respectively), 1.0% and 1.3% for Mg (for male and female, respectively), 1.5% and 2.1% for Zn (for male and female, respectively). However, few exceptions were found and included: Cu in BS4 (9.8% of its RDI), Fe in BS2 (3.4% and 1.5% of its RDI, for male and female, respectively), and Zn in BS3 (3.1% and 4.6% of its RDI for male and female, respectively) and BS7 (3.5% and 4.9% of its RDI for male and female, respectively). Only the bioaccessibility of Mn appeared to affect significantly the daily realization of its RDI, i.e., 46% (male) and 59% (female). For a better overview, the contribution of individual BSs to the RDI of Mn for male and female is presented in Figure 1.

As can be seen in Figure 1, the consumption of 1 L of BS7 and BS9 covered the RDI of Mn in 78% for male and in 99% for female. Slightly lower values, i.e., equal to 70% and 90% for male and female, respectively, were obtained for BS8. In case of BS3, results even exceeded the RDI realization of Mn by 13% for male and by 45% for female. All these results indicated that analyzed BSs are rather a poor source of Ca, Cu, Fe, Mg and Zn. However, their consumption can highly contribute to the daily Mn requirements. Nevertheless, actual nutritional benefits to human health associated with the intake of BSs can be achieved only after estimation of bioaccessibility of elements using in vitro GID, which differs from that calculated on the basis of their total concentrations. The latter approach may give untruly information. It was evident in case of the BS8, where the declared total concentration of Mn in 100 mL of this sap (0.63 mg) and the nutrient reference value (NRV, for Mn: 2 mg per day)) gave 31% realization of the NRV for this element after consumption of this 0.1 L portion. Accordingly, the intake of 1 L of BS8 per day could represent a health risk if only the total Mn concentration contained in the BS8 (declared by the producer) would be used to assess the NRV. Indeed, the value of the NRV would be as much as 310% (!). In fact, the content of Mn in the bioaccessible fraction determined here allowed us to estimate the real NRV value for Mn, i.e., 81%. Although this value was four times lower than that given by the producer, it appeared that up to 1 L of the BS8 could be safely consumed daily.

## 3. Materials and Methods

### 3.1. Samples

Bottled BSs, commercially available in Poland, were used for this study. In total, nine (*N* = 9) BSs were selected, purchased from local markets (Wroclaw, Poland) and coded as BS1–BS9. They were natural products (without any additives), pasteurized and containing high amounts of the *Betula pendula* sap, i.e., 94–100%, collected from trees growing in Ukraine (BS1, BS2, BS5, BS6), Poland (BS3), Belarus (BS8), Latvia (BS9), countries of the European Union (EU) (BS7) as well as from regions outside the UE (BS4). BS1–BS9 bottles were stored at room temperature in original packings (300–1000 mL glass bottles) and thoroughly shaken prior to sampling. After opening, they were kept in 4 °C (in a refrigerator). All sample preparations were made, however, within 2 days due to limited stability of BSs after opening their bottles.

### 3.2. Reagents and Solutions

All reagents used were of analytical grade purity. ACS grade concentrated HNO_3_ (65%, *m*/*v*) from Merck (Merck, Darmstadt, KGaA, Germany) was used for sample preparation, i.e., digestion, acidification and dilution along with acidification. A Merck Certipur^®^ (Merck, Darmstadt, KGaA, Germany) multi-elemental stock (100 mg L^−1^) ICP standard solution XVI was used to prepare simple aqueous and matrix-matched working standard solutions for calibration of an ICP OES instrument. The following ACS grade reagents (Merck, Darmstadt, Germany), i.e., HCl (37%, *m*/*v*), pepsin from porcine gastric mucosa (800–2500 units/mg of protein), pancreatin from porcine pancrease, bile salts, PIPES ((piperazine-NN-bis(2-ethane-sulfonic acid) disodium salt)), NaCl, and NaHCO_3_ were used in in vitro bioaccessibility studies. Accordingly, freshly prepared solutions of simulated gastric (SGJ) and intestinal (SIJ) juices were applied for GID of BSs. They contained 0.32% (*m*/*v*) pepsin with 0.20% (*m*/*v*) NaCl in 0.08 mol L^−1^ HCl (SGJ), and 0.40% (*m*/*v*) pancreatin with 2.5% (*m*/*v*) bile salts in 0.10 mol L^−1^ NaHCO_3_ (SIJ). A high retention cellulose dialysis tubing of 12.4 kDa MWCO (Sigma-Aldrich, St. Louis, MO, USA) was used to separate the bioaccessible fraction of studied elements from incubates of BSs. De-ionized water (18.3 MΩ cm), obtained from a Barnstead^TM^ Thermoline D7033 EASYpure RF purification system (Barnstead, NH, USA), was used throughout.

### 3.3. Instrumentation

Determination of total concentrations of studied elements, i.e., As, Ca, Cd, Cr, Cu, Fe, Mg, Mn, Ni, Pb, Sr and Zn, was made using an Agilent 720 (Agilent Technologies Inc., Santa Clara, CA, USA) bench-top simultaneous ICP OES spectrometer of the axially viewed Ar plasma, equipped with a 4-channel peristaltic pump, a high-resolution Echelle-type polychromator with temperature-controlled optics and a VistaChip II CCD detector. A concentric OneNeb nebulizer (Agilent Technologies Inc., Santa Clara, CA, USA) and a single-pass glass cyclonic spray chamber (Agilent Technologies Inc., Santa Clara, CA, USA) were applied to introduce sample and standard solutions by pneumatic nebulization (Agilent Technologies Inc., Santa Clara, CA, USA). To sustain and operate the plasma, a standard, one-piece quartz torch (2.4 mm ID injector tube) was used. The operating instrument settings were as follows: the RF power—1.2 kW, gas flow rates—15.0 (plasma), 1.5 (auxiliary) and 0.75 L min^−1^ (nebulizing), the sample flow rate—0.75 mL min^−1^ and wastes drainage rate—1.5 mL min^−1^, instrument stabilization and sample uptake delays—15 s, rinse time—10 s and replicate (*n* = 3) read time—1 s. The following emission lines (I and II denote atomic and ionic lines, respectively) were selected for measurements: As I 193.7 nm, Ca II 315.8 nm, Cd II 214.4 nm, Cr II 267.7 nm, Cu I 327.3 nm, Fe II 238.2, Mg I 285.2 nm, Mn II 257.6 nm, Ni II 231.6 nm, Pb II 220.3 nm, Sr II 407.7 nm, and Zn I 213.8 nm. Mean (*n* = 3) background-corrected intensities of these lines (using a fitted mode with 7 points per a line profile for correction) was taken for measurements. To quantify all studied elements, 9-point external calibration curves were recorded using simple aqueous and matrix-matched standard solutions within the analyte concentration range of 0.01–5.0 µg mL^−1^.

Samples were digested with the aid of a Multiwave PRO microwave reaction system (Anton Paar GmbH, Graz, Austria), equipped with a 24HVT50 rotor with 50 mL PTFE-TFM pressure-activated-venting vessels.

### 3.4. Sample Preparation Procedures Prior to Analysis

#### 3.4.1. Total Content of Elements

Six different sample preparation procedures of BSs, including digestion—reference (P1) and non-digestion—alternative (P2–P6) procedures, were tested prior to their multi-element analysis by ICP OES. All tests were performed using one selected BS, i.e., BS9. In case of digestive sample preparation procedure (P1), microwave-assisted closed-vessel digestion was applied. Alternative sample preparation procedures were based on acidification with HNO_3_ (P2, P3), dilution with low concentrated HNO_3_ (P4, P5) and no treatment (P6). Accordingly:

Microwave-assisted closed-vessel digestion (P1, reference procedure): portions of the BS9 (5.0 g) were weighed into PTFE vessels and poured with 5.0 mL of concentrated HNO_3_. Vessels were closed, shielded, inserted into the rotor and subjected to a 6-step microwave-assisted heating program with a maximum temperature of 190 °C for 60 min, i.e.,: step 1 (ramp): 90 °C, 5 min; step 2 (hold): 90 °C, 15 min; step 3 (ramp): 130 °C, 5 min; step 4 (hold): 130 °C, 15 min; step 5 (ramp): 190 °C, 5 min; step 6 (hold): 190 °C, 15 min. After cooling and opening vessels, resulting sample remnants were quantitatively transferred into 30-mL polypropylene (PP) screw-capped containers (Equimed, Kraków, Poland) and made up with deionized water to 20.0 g.

Acidification with HNO_3_ (P2, P3): portions of the BS9 (20.0 g) were weight into 30-mL PP screw-capped containers and acidified by adding appropriate volumes of concentrated HNO_3_ to reach its final concentrations of 1% (*v*/*v*) (P2) and 5% (*v*/*v*) (P3).

Dilution with low concentrated HNO_3_ (P4, P5): portions of the BS9 (10.0 g) were weight into 30-mL PP screw-capped containers and diluted 1:1 with 2% (*v*/*v*) and 10% (*v*/*v*) HNO_3_ solutions.

Direct analysis (P6): portions of the BS9 (10.0 g) were placed into 10-mL PP tubes and analyzed directly without any pre-treatment.

Until measurements, prepared sample solutions were refrigerated at 4 °C. Three parallel (*n* = 3) samples were prepared for every analysis. Along with each set of digested, acidified and diluted samples, respective procedural blanks were simultaneously run (using deionized water instead of the BS) and considered in final results. Blanks were also used as diluents of matrix (HNO_3_)-matched standard solutions for given sample preparation procedures. In case of direct analysis (P6), just deionized water was used as blank, hence, ICP OES measurements were made against simple aqueous standards. Except for Ca and Mg, concentrations of remaining elements were determined in undiluted sample solutions. In case of measurements of Ca and Mg, prepared sample solutions were appropriately diluted, i.e., 5–20-fold.

#### 3.4.2. Bioaccessible Fraction–In Vitro Gastrointestinal Digestion Procedure

Determination of bioaccessibility of elements in BSs was made with the aid of in vitro GID. A two-step procedure with SGJ and SIJ solutions were used to simulate GID as detailed before [15,16]. In brief, for in vitro GID, samples (aliquots (20.0 g, *n* = 3) of each BSs and water (treated as a blank)) were placed in 50-mL PP tubs, adjusted to pH 2.0 with a 6.0 mol L^−1^ HCl solution and filled with 3.0 mL of the SGJ solution to simulate gastric digestion. Samples were incubated in a temperature-controlled, shaking water bath at 37 °C with agitation (150 rpm) for 2 h before the enzymatic reaction was stopped by placing tubes into an ice-bath for 10 min. After this, 5.0 mL of the SIJ solution was added to simulate intestinal digestion. Dialysis membrane tubings with 20 mL of a PIPES solution (0.15 mol L^−1^, pH 7.5 adjusted with HCl) were placed inside these tubes, and incubation was continued (37 °C, agitation 150 rpm) for next 2 h. Then, enzymatic reaction was stopped again (ice-bath, 10 min). Next, contents of dialysis membrane tubings (abbreviated as dialyzable or bioaccessible fraction) and residual solutions of tubes (abbreviated as non-dialyzable fraction, or residue) were transferred to 30-mL PP containers, and analyzed directly by ICP OES. External calibration with matrix-matched standard solutions (in reference to components of SGJ and SIJ), prepared based on respective blanks, was used for this purpose, covering the concentration range within 0.01–5.0 µg mL^−1^.

## 4. Conclusions

A special care for selection of proper sample preparation before multi-element analysis by ICP OES is required and, as shown in the present work, is a critical factor that affects trueness of final results. For the first time, a fully validated simplified ICP OES-based method, involving no treatment of respective samples, and applicable for precise (RSD < 5%), true (98.0–104% as recoveries), and sensitive (LODs at 0.020–5.8 ng mL^−1^) determination of 12 elements in bottled BSs, was proposed. In comparison to the traditional approach based on wet digestion, it completely omitted the sample preparation step, making the proposed method simpler, cheaper, safer, and faster. In fact, it permitted multi-element analysis of the BS within ~1 min per sample, hence allowed to quickly evaluate its mineral composition (total concentrations of elements) that is essential in monitoring its quality. The method was successfully applied to analysis of several commercially available bottled BSs in terms of their quality and nutritional value, the latter realized through determination of bioaccessibility of elements from BSs by means of the in vitro GID procedure. Accordingly, it was concluded that all analyzed BSs were safe, i.e., contained mainly Mg and Ca, and small quantities of Mn, Zn, Cu, Zn, and Fe, whereas toxic components as As, Cd, Ni, and Pb were present at levels lower than their LODs. Results of GID indicated that the bioaccessibility of nutritious elements from BSs was lower than 40%. In view of this, it was revealed that drinking daily 1 L of BSs covered to a small degree (<2.5%) respective RDIs of Ca, Cu, Fe, Mg, and Zn, indicating that the analyzed BSs were rather a poor source of the aforementioned elements. The consumption of BSs contributed highly only to the RDI of Mn.

## Figures and Tables

**Table 1:** Limits of detection (LODs) achievable with inductively coupled plasma optical emission spectrometry (ICP OES) combined with different sample preparation procedures, i.e., reference (P1) and alternative (P2–P6) procedures.

	LOD ^a^, ng mL^−1^						LOD ^b^, ng mL^−1^					
	P1	P2	P3	P4	P5	P6	P1	P2	P3	P4	P5	P6
As	6.2	6.4	6.4	6.5	6.1	5.8	25	6.5	7.1	13	12	5.8
Ca	2.2	4.8	2.1	4.0	1.8	1.5	8.8	4.9	2.3	8.0	3.6	1.5
Cd	0.57	0.49	0.65	0.57	0.48	0.31	2.3	0.50	0.72	1.1	0.96	0.31
Cr	1.4	0.75	0.80	0.69	0.68	0.74	5.6	0.76	0.89	1.4	1.4	0.74
Cu	0.84	0.90	0.69	0.86	0.66	0.68	3.4	0.92	0.77	1.7	1.3	0.68
Fe	5.2	0.78	2.0	0.59	1.9	0.52	21	0.80	2.2	1.2	3.8	0.52
Mg	0.69	0.52	0.64	0.44	0.42	0.14	2.8	0.53	0.71	0.88	0.84	0.14
Mn	0.13	0.090	0.19	0.050	0.17	0.09	0.52	0.090	0.21	0.10	0.34	0.09
Ni	2.4	3.0	3.3	3.4	2.8	1.5	9.6	3.1	3.7	6.8	5.6	1.5
Pb	3.7	4.0	5.0	3.8	4.8	3.1	15	4.1	5.6	7.6	9.6	3.1
Sr	0.080	0.060	0.060	0.050	0.060	0.020	0.32	0.060	0.070	0.10	0.12	0.020
Zn	0.40	0.24	0.14	0.22	0.12	0.08	1.6	0.24	0.15	0.44	0.24	0.080

P1: microwave-assisted closed-vessel digestion in concentrated HNO_3_. P2: acidification to 1% (*v*/*v*) with concentrated HNO_3_. P3: acidification to 5% (*v*/*v*) with concentrated HNO_3_. P4: 2-fold (1:1, *w*/*w*) dilution with 2% (*v*/*v*) HNO_3_. P5: 2-fold (1:1, *w*/*w*) dilution with 10% (*v*/*v*) HNO_3_. P6: direct analysis (no pre-treatment). ^a^ In measured sample solution. ^b^ In original sample (final dilution factors and sample portions used in respective sample preparation procedures (P1–P6) was included).

**Table 2:** Concentrations of elements determined by ICP OES in the selected birch sap (BS9) prepared before measurements using different sample preparation procedures.

	Concentrations ^a^, µg mL^−1^					
	P1	P2	P3	P4	P5	P6
As	<25 ^b^	<6.5 ^b^	<7.1 ^b^	<13 ^b^	< 12 ^b^	<5.8 ^b^
Ca	62.6 (3.8)	56.8 (1.1)	52.6 (0.38)	56.1 (2.5)	54.0 (1.1)	62.2 (1.8)
Cd	<2.3 ^b^	<0.50 ^b^	<0.72 ^b^	<1.1 ^b^	< 0.96 ^b^	<0.31 ^b^
Cr ^c^	<5.6 ^b^	2.83 (3.6)	2.73 (11)	4.98 (16)	2.76 (14)	5.06 (2.0)
Cu ^c^	8.30 (3.6)	5.05 (2.0)	4.51 (2.3)	5.18 (3.0)	4.84 (2.1)	8.21 (1.2)
Fe	0.885 (4.2)	0.778 (1.9)	0.770 (1.6)	0.798 (1.5)	0.774 (1.8)	0.883 (4.1)
Mg	14.7 (0.68)	13.0 (0.77)	12.3 (0.81)	13.2 (3.0)	12.8 (0.78)	14.6 (0.68)
Mn	4.36 (3.7)	3.77 (0.80)	3.62 (0.28)	4.29 (2.6)	3.72 (1.3)	4.44 (1.1)
Ni	<9.6 ^b^	<3.1 ^b^	<3.7 ^b^	<6.8 ^b^	<5.6 ^b^	<1.5 ^b^
Pb	<15 ^b^	<4.1 ^b^	<5.6 ^b^	<7.6 ^b^	<9.6 ^b^	<3.1 ^b^
Sr	0.178 (2.2)	0.143 (2.1)	0.143 (2.1)	0.167 (3.6)	0.156 (1.9)	0.177 (2.3)
Zn	1.16 (3.4)	1.00 (2.0)	0.948 (0.95)	1.08 (3.7)	0.962 (2.5)	1.15 (2.6)

P1: microwave-assisted closed-vessel digestion in concentrated HNO_3_. P2: acidification to 1% (*v*/*v*) with concentrated HNO_3_. P3: acidification to 5% (*v*/*v*) with concentrated HNO_3_. P4: 2-fold (1:1, *w*/*w*) dilution with 2% (*v*/*v*) HNO_3_. P5: 2-fold (1:1, *w*/*w*) dilution with 10% (*v*/*v*) HNO_3_. P6: direct analysis (no pre-treatment). ^a^ Means (*n* = 3) with relative standard deviations (%RSDs) in brackets. ^b^ Below the limit of detection (LOD, ng mL^−1^) in the original sample (final dilution factors and sample portions used in respective sample preparation procedures (P1–P6) was included). ^c^ Concentration in ng mL^−1^.

**Table 3:** Calculated values of the *F*-test (*F*_calculated_) and the *t*-test (|*t*_calculated_|) for comparison of standard deviations of mean concentrations of elements and these mean concentrations, respectively, determined by ICP OES in the selected birch sap (BS9) prepared before measurements using different alternative sample preparation procedures (P2–P6) against the reference sample preparation procedure (P1).

	*F*_calculated_^a^					|*t*_calculated_| ^b^				
	P2/P1	P3/P1	P4/P1	P5/P1	P6/P1	P2/P1	P3/P1	P4/P1	P5/P1	P6/P1
Ca	16.00	144.00	2.94	16.00	4.76	4.061	5.872 ^c^	4.052	6.021	0.262
Cu	9.00	9.00	2.25	9.00	9.00	17.801	20.759	14.988	18.951	0.548
Fe	6.08	9.51	9.51	6.98	1.06	4.642	5.121	3.874	4.860	0.067
Mg	1.00	1.00	16.00	1.00	1.00	20.821	29.394	6.301	23.270	1.225
Mn	28.44	256.00	2.12	10.24	10.24	5.126 ^c^	6.528 ^c^	0.624	6.613	0.827
Sr	16.00	1.78	2.25	1.78	1.00	8.822	12.1244	2.642	7.621	0.306
Zn	4.00	19.75	1.00	2.78	1.78	6.197	7.312 ^c^	2.449	7.352	0.346

Significant differences are underlined. P2: acidification to 1% (*v*/*v*) with concentrated HNO_3_. P3: acidification to 5% (*v*/*v*) with concentrated HNO_3_. P4: 2-fold (1:1, *w*/*w*) dilution with 2% HNO_3_. P5: 2-fold (1:1, *w*/*w*) dilution with 10% (*v*/*v*) HNO_3_. P6: direct analysis (no pre-treatment). ^a^ The critical value of the *F*-test (*F*_critical_): 19.00 (*p* = 0.05). ^b^ The critical value of the *t*-test (*t*_critical_): 2.776 (*p* = 0.05). ^c^ The case when *F*_calculated_ > *F*_critical_. The Cochran-Cox *C*-test with the critical value (*C_critical_*): 4.303 (*p* = 0.05) was used.

**Table 4:** Recoveries of elements in the selected birch sap (BS9) prepared prior to ICP OES analysis by using reference (P1) and alternative (P2–P6) sample preparation procedures.

	Addition	Recovery ^a^, %					
		P1	P2	P3	P4	P5	P6
As	0.010	106 ± 4	103 ± 3	119 ± 5	108 ± 6	86.6 ± 3.6	103 ± 3
	0.025	103 ± 3	105 ± 1	116 ± 4	106 ± 3	90.2 ± 2.2	102 ± 2
	0.050	102 ± 3	109 ± 1	112 ± 3	104 ± 1	95.3 ± 2.6	102 ± 2
Ca	0.10	99.0 ± 1.0	109 ± 1	107 ± 1	113 ± 1	110 ± 1	101 ± 1
	0.25	99.8 ± 2.2	109 ± 1	106 ± 1	112 ± 2	110 ± 1	99.8 ± 0.2
	0.50	103 ± 1	110 ± 1	106 ± 2	114 ± 1	110 ± 1	102 ± 1
Cd	0.010	105 ± 3	106 ± 4	108 ± 3	104 ± 4	108 ± 3	103 ± 2
	0.025	102 ± 2	109 ± 4	112 ± 3	106 ± 3	111 ± 2	102 ± 1
	0.050	102 ± 2	112 ± 3	117 ± 2	108 ± 3	117 ± 1	99.8 ± 1.1
Cr	0.010	96.9 ± 0.1	92.5 ± 3.3	90.2 ± 3.6	101 ± 2	91.6 ± 1.7	100 ± 2
	0.025	104 ± 4	105 ± 5	94.3 ± 2.1	101 ± 4	93.7 ± 3.3	101 ± 3
	0.050	101 ± 3	129 ± 2	116 ± 4	103 ± 2	116 ± 1	103 ± 1
Cu	0.010	99.1 ± 0.3	93.4 ± 1.6	95.1 ± 1.5	90.5 ± 1.3	91.6 ± 1.7	99.7 ± 0.5
	0.025	102 ± 2	94.4 ± 1.1	95.4 ± 2.1	110 ± 1	93.9 ± 1.7	104 ± 2
	0.050	101 ± 2	97.6 ± 0.4	118 ± 1	124 ± 2	94.9 ± 0.9	103 ± 1
Fe	0.10	105 ± 9	97.9 ± 0.1	87.5 ± 0.2	82.9 ± 0.1	103 ± 3	100 ± 1
	0.25	106 ± 5	104 ± 2	90.3 ± 1.1	88.9 ± 0.8	98.9 ± 2.1	103 ± 1
	0.50	102 ± 5	100 ± 1	91.4 ± 0.9	91.3 ± 0.6	99.5 ± 0.8	102 ± 1
Mg	0.10	105 ± 1	92.0 ± 1.0	89.6 ± 0.5	83.6 ± 0.2	76.6 ± 0.9	98.0 ± 0.3
	0.25	107 ± 2	92.9 ± 0.5	88.3 ± 0.6	83.9 ± 1.5	76.7 ± 0.2	98.3 ± 0.2
	0.50	109 ± 1	93.4 ± 0.6	89.4 ± 1.5	85.2 ± 0.8	77.6 ± 0.3	99.7 ± 0.5
Mn	0.10	98.7 ± 0.6	95.1 ± 2.1	93.3 ± 2.3	97.2 ± 4.9	94.4 ± 1.9	99.1 ± 1.0
	0.25	103 ± 2	96.3 ± 1.8	93.9 ± 2.5	101 ± 1	95.0 ± 2.1	100 ± 1
	0.50	102 ± 1	96.4 ± 1.6	95.3 ± 1.8	100 ± 1	95.8 ± 1.7	101 ± 1
Ni	0.010	98.9 ± 2.4	97.1 ± 3.3	96.4 ± 3.3	98.5 ± 3.1	96.8 ± 3.2	99.2 ± 2.1
	0.025	99.2 ± 2.1	98.3 ± 2.4	108 ± 3	103 ± 2	106 ± 2	101 ± 2
	0.050	102 ± 2	105 ± 2	111 ± 2	108 ± 2	107 ± 2	101 ± 1
Pb	0.010	98.8 ± 0.7	93.5 ± 1.3	89.9 ± 2.7	96.2 ± 0.6	90.8 ± 1.7	98.6 ± 0.8
	0.025	99.2 ± 0.4	94.6 ± 1.0	91.0 ± 1.5	96.5 ± 0.7	92.1 ± 1.5	100 ± 1
	0.050	99.5 ± 1.0	95.9 ± 0.8	93.0 ± 2.8	96.9 ± 1.3	93.6 ± 1.2	102 ± 1
Sr	0.10	98.3 ± 0.8	95.0 ± 1.6	121 ± 4	97.0 ± 1.4	89.1 ± 0.5	99.6 ± 1.2
	0.25	100 ± 2	96.2 ± 1.9	117 ± 3	101 ± 1	95.3 ± 1.6	101 ± 1
	0.50	102 ± 1	106 ± 1	117 ± 3	104 ± 3	106 ± 2	102 ± 1
Zn	0.10	98.4 ± 0.1	103 ± 1	94.2 ± 2.2	106 ± 2	93.6 ± 1.5	104 ± 1
	0.25	104 ± 1	105 ± 1	95.1 ± 1.9	100 ± 1	96.7 ± 0.1	101 ± 1
	0.50	103 ± 1	107 ± 1	96.5 ± 2.4	100 ± 1	96.9 ± 1.3	98.7 ± 0.2

P1: microwave-assisted closed-vessel digestion in concentrated HNO_3_. P2: acidification to 1% (*v*/*v*) with concentrated HNO_3_. P3: acidification to 5% (*v*/*v*) with concentrated HNO_3_. P4: 2-fold (1:1, *w*/*w*) dilution with 2% (*v*/*v*) HNO_3_. P5: 2-fold (1:1, *w*/*w*) dilution with 10% (*v*/*v*) HNO_3_. P6: direct analysis (no pre-treatment). ^a^ Recovery of added elements into respective sample solutions achieved for ICP OES. Mean values (*n* = 3) ± standard deviations (SDs).

**Table 5:** Results of multi-element analysis of different birch saps (BS1–BS9) by ICP OES with no sample treatment.

	Concentration ^a^, µg mL^−1^									
	BS1	BS2	BS3	BS4	BS5	BS6	BS7	BS8	BS9	Mean ^b^
As	<5.8 ^c^	<5.8 ^c^	<5.8 ^c^	<5.8 ^c^	<5.8 ^c^	<5.8 ^c^	<5.8 ^c^	<5.8 ^c^	<5.8 ^c^	<5.8 ^c^
Ca	52.0 (0.62)	43.7 (0.40)	59.1 (0.93)	49.4 (0.77)	46.5 (0.81)	45.7 (0.91)	43.9 (0.57)	50.2 (0.87)	62.2 (1.8)	50.3 (13.0)
Cd	<0.31 ^c^	<0.31 ^c^	<0.31 ^c^	<0.31 ^c^	<0.31 ^c^	<0.31 ^c^	<0.31 ^c^	<0.31 ^c^	<0.31 ^c^	<0.31 ^c^
Cr ^d^	2.45 (4.7)	*<0.74* ^c^	10.1 (4.3)	8.50 (1.7)	*<0.74* ^c^	*<0.74* ^c^	3.47 (3.0)	7.23 (2.4)	5.06 (2.0)	4.34 (82.6)
Cu ^d^	92.1 (0.22)	64.3 (1.3)	12.4 (4.2)	313 (1.2)	143 (1.1)	74.2 (0.70)	81.9 (0.16)	29.6 (2.4)	8.21 (2.1)	91.0 (103)
Fe	0.260 (1.1)	1.81 (0.84)	1.22 (0.82)	0.815 (0.32)	0.255 (0.68)	0.556 (0.69)	0.613 (1.4)	1.84 (0.63)	0.883 (4.1)	0.917 (65.0)
Mg	11.5 (0.87)	8.66 (0.55)	16.8 (0.91)	10.2 (1.1)	10.6 (1.1)	10.3 (1.1)	11.0 (0.91)	15.3 (0.75)	14.6 (0.68)	12.1 (22.8)
Mn	0.561 (1.1)	0.523 (0.58)	7.73 (0.65)	3.07 (0.99)	0.689 (0.33)	0.514 (0.78)	5.88 (0.43)	6.28 (1.1)	4.44 (1.1)	3.30 (87.3)
Ni	<1.5 ^c^	<1.5 ^c^	<1.5 ^c^	<1.5 ^c^	<1.5 ^c^	<1.5 ^c^	<1.5 ^c^	<1.5 ^c^	<1.5 ^c^	<1.5 ^c^
Pb	<3.1 ^c^	<3.1 ^c^	<3.1 ^c^	<3.1 ^c^	<3.1 ^c^	<3.1 ^c^	<3.1 ^c^	<3.1 ^c^	<3.1 ^c^	<3.1 ^c^
Sr	0.208 (0.53)	0.170 (0.74)	0.254 (1.1)	0.189 (1.2)	0.185 (0.98)	0.183 (0.86)	0.126 (0.25)	0.192 (1.4)	0.177 (2.3)	0.187 (18.0)
Zn	0.137 (0.43)	0.083 (0.48)	1.06 (0.55)	0.443 (0.90)	0.104 (1.0)	0.0740 (0.59)	1.49 (0.39)	0.947 (0.90)	1.15 (2.6)	0.610 (90.8)

^a^ Mean values (*n* = 3) with relative standard deviations (%RSDs) in brackets. ^b^ Average values for all BSs with coefficients of variance (%CV) in brackets. ^c^ Below the limit of detection (LOD, ng mL^−1^). ^d^ Concentration in ng mL^−1^.

**Table 6:** Percentage contributions of the bioaccessible fraction of elements from all analyzed birch saps (BS1–BS9).

	Contribution of the Bioaccessible Fraction ^a^, %								
	BS1	BS2	BS3	BS4	BS5	BS6	BS7	BS8	BS9
Ca	38.5 ± 1.3	33.9 ± 0.7	38.6 ± 1.3	36.0 ± 0.5	36.1 ± 0.7	35.9 ± 1.3	36.4 ± 0.8	37.3 ± 1.2	44.9 ± 1.4
Cu	22.8 ± 1.1	20.3 ± 1.0	25.0 ± 1.5	28.1 ± 0.7	21.0 ± 1.0	17.6 ± 1.2	18.3 ± 0.8	23.3 ± 1.5	25.0 ± 1.8
Fe	19.2 ± 0.9	15.2 ± 0.7	16.0 ± 0.4	15.5 ± 0.4	13.7 ± 0.7	14.4 ± 0.6	19.7 ± 0.6	14.5 ± 0.3	13.3 ± 0.3
Mg	32.8 ± 0.9	37.1 ± 1.0	42.0 ± 0.6	30.2 ± 0.8	31.4 ± 1.4	32.5 ± 1.5	37.7 ± 1.2	30.5 ± 0.9	41.4 ± 1.2
Mn	33.5 ± 1.1	32.5 ± 1.0	33.8 ± 1.6	35.8 ± 0.8	27.9 ± 0.9	23.0 ± 0.6	30.3 ± 0.4	25.8 ± 0.3	40.3 ± 0.4
Sr	37.5 ± 1.2	37.1 ± 1.1	40.6 ± 0.4	42.9 ± 1.6	32.4 ± 1.4	28.4 ± 1.4	39.7 ± 2.0	33.3 ± 1.4	40.1 ± 1.3
Zn	29.9 ± 1.3	25.3 ± 1.0	34.3 ± 1.5	24.8 ± 1.0	33.7 ± 0.4	28.4 ± 1.0	26.1 ± 0.7	26.9 ± 1.0	24.0 ± 1.0

^a^ Mean values (*n* = 3) ± standard deviations (SDs).

**Table 7:** A mass balance study for gastrointestinal digestion (GID) and mean concentrations of elements determined by ICP OES in dialyzable and non-dialyzable fractions of analyzed birch saps (BS1–BS9).

	BS1	BS2	BS3	BS4	BS5	BS6	BS7	BS8	BS9	Mean ^a^
Ca										
C_t_^b^	52.0 ± 0.3	43.7 ± 0.2	59.1 ± 0.5	49.4 ± 0.4	46.5 ± 0.4	45.7 ± 0.4	43.9 ± 0.2	50.2 ± 0.4	62.2 ± 1.1	50.3 ± 6.5
A: dialysate ^c^	20.0 ± 0.7	14.8 ± 0.3	22.8 ± 0.8	17.8 ± 0.3	16.8 ± 0.3	16.4 ± 0.6	16.0 ± 0.4	18.7 ± 0.6	27.9 ± 0.8	19.0 ± 4.1
B: non-dialyzate ^d^	33.0 ± 0.8	30.6 ± 0.6	38.2 ± 1.3	32.6 ± 0.4	30.5 ± 0.5	30.7 ± 1.1	28.9 ± 0.6	32.8 ± 1.2	35.2 ± 1.0	32.5 ± 2.8
Sum (A and B) ^e^	53.0 ± 1.5	45.4 ± 0.9	61.0 ± 2.0	50.4 ± 0.7	47.3 ± 0.8	47.1 ± 1.7	44.9 ± 1.0	51.5 ± 1.8	63.1 ± 1.9	51.5 ± 6.6
Agreement ^f^	101.9 ± 2.9	103.9 ± 2.0	103.2 ± 3.3	102.0 ± 1.3	101.7 ± 1.8	103.1 ± 3.6	102.3 ± 2.3	102.6 ± 3.4	101.4 ± 2.8	102.5 ± 0.8
Cu										
C_t_^b^	0.092 ± 0.001	0.064 ± 0.001	0.012 ± 0.001	0.313 ± 0.004	0.143 ± 0.002	0.074 ± 0.001	0.082 ± 0.001	0.030 ± 0.001	0.008 ± 0.001	0.091 ± 0.094
A: dialysate ^c^	0.021 ± 0.001	0.013 ± 0.001	0.003 ± 0.001	0.088 ± 0.002	0.030 ± 0.001	0.013 ± 0.001	0.015 ± 0.001	0.007 ± 0.001	0.002 ± 0.001	0.021 ± 0.026
B: non-dialyzate ^d^	0.070 ± 0.002	0.054 ± 0.002	0.010 ± 0.001	0.242 ± 0.004	0.115 ± 0.006	0.066 ± 0.003	0.070 ± 0.001	0.022 ± 0.001	0.006 ± 0.001	0.073 ± 0.072
Sum (A and B) ^e^	0.091 ± 0.004	0.067 ± 0.003	0.013 ± 0.001	0.330 ± 0.007	0.145 ± 0.007	0.079 ± 0.004	0.085 ± 0.002	0.029 ± 0.002	0.008 ± 0.001	0.094 ± 0.098
Agreement ^f^	98.8 ± 4.1	104.7 ± 4.7	108.3 ± 5.6	105.4 ± 2.3	101.4 ± 4.9	106.8 ± 5.4	103.7 ± 2.8	96.7 ± 5.5	100.0 ± 7.1	102.8 ± 3.9
Fe										
C_t_^b^	0.260 ± 0.003	1.81 ± 0.02	1.22 ± 0.01	0.815 ± 0.003	0.255 ± 0.002	0.556 ± 0.004	0.613 ± 0.009	1.84 ± 0.01	0.883 ± 0.036	0.917 ± 0.596
A: dialysate ^c^	0.050 ± 0.002	0.275 ± 0.013	0.195 ± 0.005	0.126 ± 0.003	0.035 ± 0.002	0.080 ± 0.003	0.121 ± 0.004	0.266 ± 0.005	0.117 ± 0.003	0.140 ± 0.087
B: non-dialyzate ^d^	0.215 ± 0.008	1.58 ± 0.07	1.07 ± 0.05	0.692 ± 0.037	0.216 ± 0.008	0.492 ± 0.016	0.500 ± 0.014	1.59 ± 0.05	0.794 ± 0.039	0.794 ± 0.523
Sum (A and B) ^e^	0.265 ± 0.011	1.86 ± 0.09	1.26 ± 0.05	0.818 ± 0.031	0.251 ± 0.011	0.572 ± 0.014	0.621 ± 0.018	1.86 ± 0.05	0.911 ± 0.032	0.935 ± 0.609
Agreement ^f^	101.9 ± 4.2	102.5 ± 4.9	103.7 ± 3.6	100.4 ± 3.8	98.4 ± 4.1	102.9 ± 3.8	101.3 ± 2.8	100.9 ± 2.6	103.2 ± 3.5	101.7 ± 1.6
Mg										
C_t_^b^	11.5 ± 0.1	8.66 ± 0.05	16.8 ± 0.2	10.2 ± 0.1	10.6 ± 0.1	10.3 ± 0.1	11.0 ± 0.1	15.3 ± 0.1	14.6 ± 0.1	12.1 ± 2.8
A: dialysate ^c^	3.77 ± 0.11	3.21 ± 0.09	7.06 ± 0.11	3.08 ± 0.09	3.33 ± 0.15	3.35 ± 0.15	4.15 ± 0.14	4.66 ± 0.14	6.05 ± 0.17	4.30 ± 1.40
B: non-dialyzate ^d^	8.01 ± 0.14	5.81 ± 0.13	10.0 ± 0.1	7.23 ± 0.11	7.31 ± 0.19	7.06 ± 0.16	7.18 ± 0.15	10.8 ± 0.1	8.72 ± 0.12	8.01 ± 1.57
Sum (A and B) ^e^	11.8 ± 0.3	9.02 ± 0.22	17.1 ± 0.2	10.3 ± 0.2	10.6 ± 0.4	10.4 ± 0.4	11.3 ± 0.3	15.5 ± 0.3	14.8 ± 0.3	12.3 ± 2.8
Agreement ^f^	102.4 ± 2.4	104.2 ± 2.6	101.5 ± 1.4	101.1 ± 2.2	100.4 ± 3.5	101.1 ± 3.4	103.0 ± 2.8	101.0 ± 2.2	101.2 ± 2.1	101.8 ± 1.2
Mn										
C_t_^b^	0.561 ± 0.006	0.523 ± 0.003	7.73 ± 0.05	3.07 ± 0.03	0.689 ± 0.002	0.514 ± 0.004	5.88 ± 0.02	6.28 ± 0.07	4.44 ± 0.05	3.30 ± 2.89
A: dialysate ^c^	0.188 ± 0.006	0.170 ± 0.005	2.61 ± 0.11	1.10 ± 0.01	0.192 ± 0.006	0.118 ± 0.003	1.78 ± 0.02	1.62 ± 0.02	1.79 ± 0.02	1.06 ± 0.93
B: non-dialyzate ^d^	0.383 ± 0.011	0.358 ± 0.011	5.18 ± 0.04	2.00 ± 0.04	0.515 ± 0.014	0.399 ± 0.011	4.16 ± 0.04	4.77 ± 0.05	2.55 ± 0.03	2.26 ± 2.01
Sum (A and B) ^e^	0.571 ± 0.017	0.528 ± 0.016	7.79 ± 0.21	3.10 ± 0.06	0.707 ± 0.021	0.517 ± 0.015	5.94 ± 0.06	6.39 ± 0.08	4.34 ± 0.05	3.32 ± 2.90
Agreement ^f^	101.8 ± 3.1	101.0 ± 3.1	100.8 ± 2.7	101.0 ± 2.0	102.6 ± 3.1	100.6 ± 2.9	101.0 ± 1.1	101.8 ± 1.2	97.7 ± 1.2	100.9 ± 1.3
Sr										
C_t_^b^	0.208 ± 0.001	0.170 ± 0.001	0.254 ± 0.003	0.189 ± 0.002	0.185 ± 0.002	0.183 ± 0.002	0.126 ± 0.001	0.192 ± 0.003	0.177 ± 0.004	0.187 ± 0.034
A: dialysate ^c^	0.078 ± 0.002	0.063 ± 0.002	0.103 ± 0.001	0.081 ± 0.003	0.060 ± 0.002	0.052 ± 0.002	0.050 ± 0.003	0.064 ± 0.003	0.071 ± 0.002	0.069 ± 0.016
B: non-dialyzate ^d^	0.129 ± 0.002	0.110 ± 0.002	0.154 ± 0.003	0.115 ± 0.003	0.125 ± 0.003	0.133 ± 0.003	0.077 ± 0.001	0.132 ± 0.002	0.112 ± 0.002	0.121 ± 0.021
Sum (A and B) ^e^	0.207 ± 0.005	0.173 ± 0.004	0.257 ± 0.004	0.196 ± 0.007	0.185 ± 0.006	0.185 ± 0.007	0.127 ± 0.004	0.196 ± 0.005	0.183 ± 0.005	0.190 ± 0.034
Agreement ^f^	99.5 ± 2.4	101.8 ± 2.5	101.2 ± 1.5	103.7 ± 3.5	100.0 ± 3.4	101.1 ± 3.5	100.8 ± 3.3	102.1 ± 2.8	103.4 ± 2.7	101.5 ± 1.4
Zn										
C_t_^b^	0.137 ± 0.001	0.083 ± 0.001	1.06 ± 0.01	0.443 ± 0.004	0.104 ± 0.001	0.074 ± 0.001	1.49 ± 0.01	0.947 ± 0.009	1.15 ± 0.03	0.610 ± 0.554
A: dialysate ^c^	0.041 ± 0.0018	0.021 ± 0.001	0.364 ± 0.016	0.110 ± 0.005	0.035 ± 0.001	0.021 ± 0.001	0.389 ± 0.010	0.255 ± 0.010	0.276 ± 0.012	0.168 ± 0.153
B: non-dialyzate ^d^	0.099 ± 0.004	0.064 ± 0.001	0.739 ± 0.024	0.342 ± 0.013	0.069 ± 0.001	0.054 ± 0.002	1.11 ± 0.02	0.709 ± 0.024	0.887 ± 0.034	0.453 ± 0.413
Sum (A and B) ^e^	0.140 ± 0.006	0.085 ± 0.002	1.10 ± 0.04	0.452 ± 0.018	0.104 ± 0.001	0.075 ± 0.003	1.50 ± 0.04	0.964 ± 0.035	1.16 ± 0.05	0.621 ± 0.562
Agreement ^f^	102.2 ± 4.3	102.4 ± 2.1	104.1 ± 3.9	102.0 ± 4.0	100.0 ± 0.8	101.4 ± 3.6	100.6 ± 2.4	101.8 ± 3.7	101.1 ± 4.1	101.7 ± 1.2

^a^ Mean values (*n* = 3) ± standard deviations (SDs). ^b^ Total concentrations of elements (in µg mL^−1^) determined using the developed simplified method of multi-element analysis of birch saps by ICP OES. ^c^ Concentrations of elements (in µg mL^−1^) in the dialyzable fraction after GID procedure. ^d^ Concentrations of elements (in µg mL^−1^) in the non-dialyzable fraction after GID procedure. ^e^ Sums of concentrations of elements (in µg mL^−1^) in dialyzable and non-dialyzable fractions. ^f^ In % calculated as [(A + B)/C_t_] × 100.
